# Supplementary material for: Distinct and joint effects of low and high levels of Aβ and tau deposition on cortical thickness
Source: Neuroimage Clin. 2023 Apr 19;38:103409. doi: 10.1016/j.nicl.2023.103409 (PMC10165160; doi:10.1016/j.nicl.2023.103409)
Supplement: Supplementary data 1 [file mmc1.docx]

**Supplementary material:**


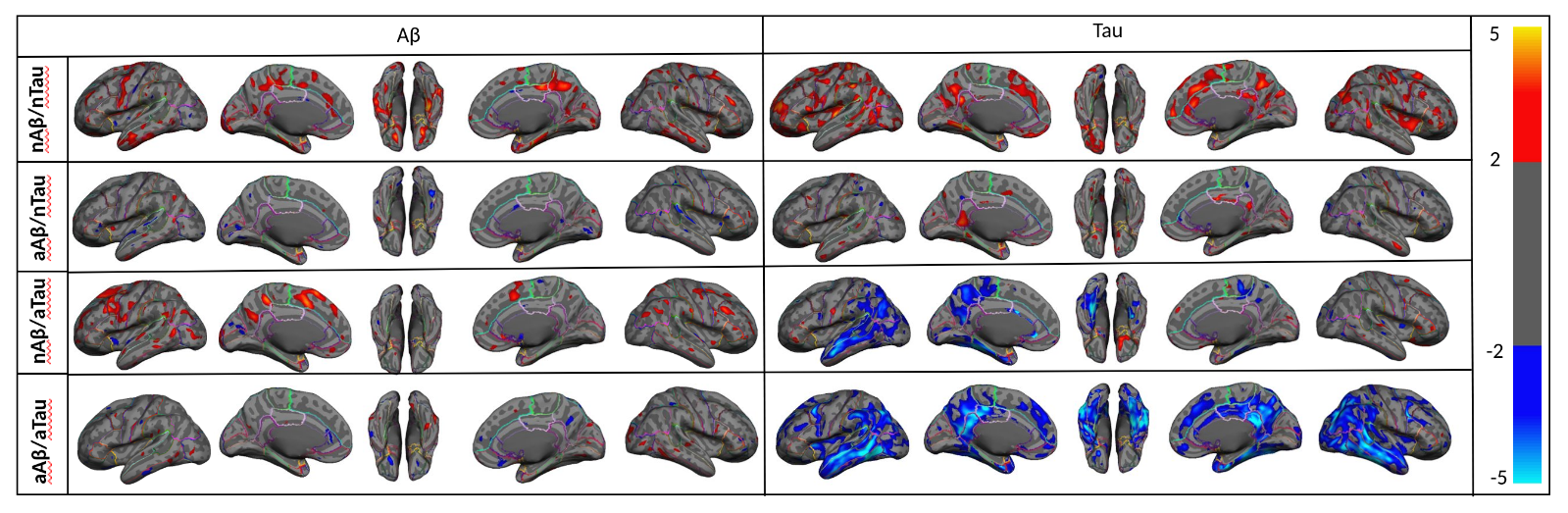


Fig. S1: Vertex-wise statistical map (t-value) of association between global Aβ (left column), global tau (right column) pathologies, and cortical thickness throughout the entire cerebral cortex obtained in four categories of participants. (First row, nAβ/nTau; second row, aAβ/nTau; third row, nAβ/aTau; and fourth row, aAβ/aTau). The t-value at each vertex is color-coded with red to yellow colors representing increasing positive t-values and blue to light blue representing decreasing negative t-values and overlaid on the semi-inflated cortical surface of the MNI152 template. The association between global Aβ, global tau pathologies, and cortical thickness was not corrected for multiple comparison corrections.


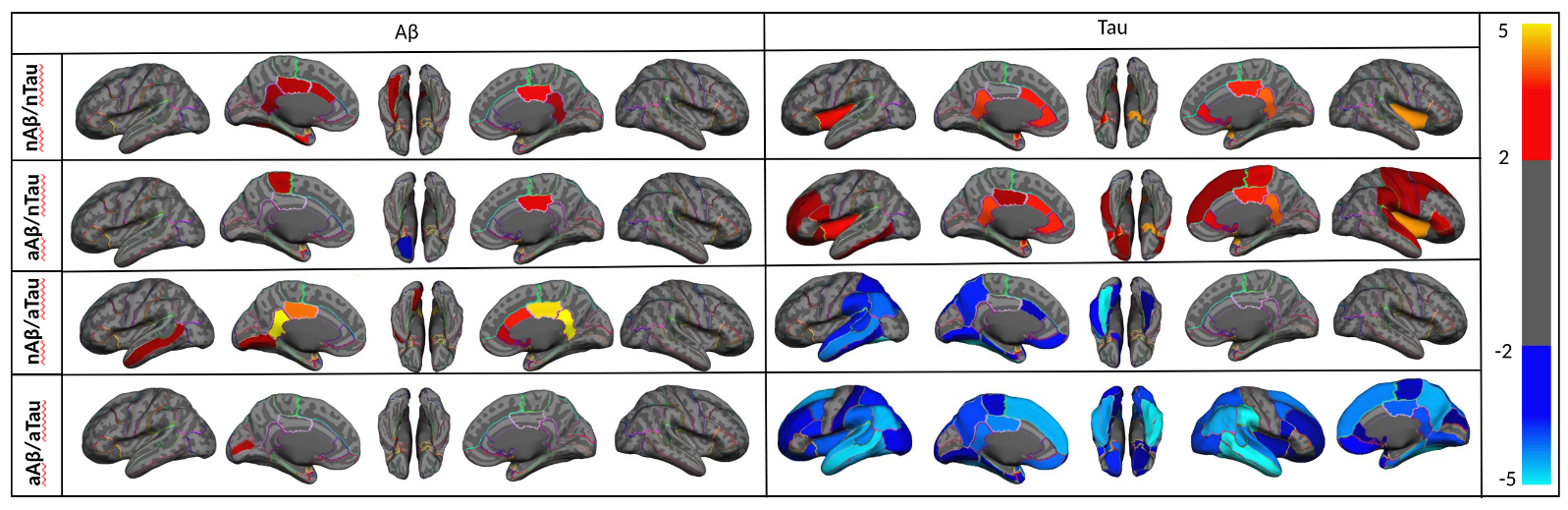


Fig. S2: Region-wise statistical map (t-value) of association between regional Aβ (left column), regional tau (right column) pathologies, and regional cortical thickness throughout cortical 68 ROIs obtained in four categories of participants (First row, nAβ/nTau; second row, aAβ/nTau; third row, nAβ/aTau; and fourth row, aAβ/aTau). The t-value at each region is color-coded with red to yellow colors representing increasing positive t-values and blue to light blue representing decreasing negative t-values and overlaid on the semi-inflated cortical surface of the MNI152 template. Association between regional Aβ, tau pathologies, and cortical thickness was not corrected for family-wise errors.


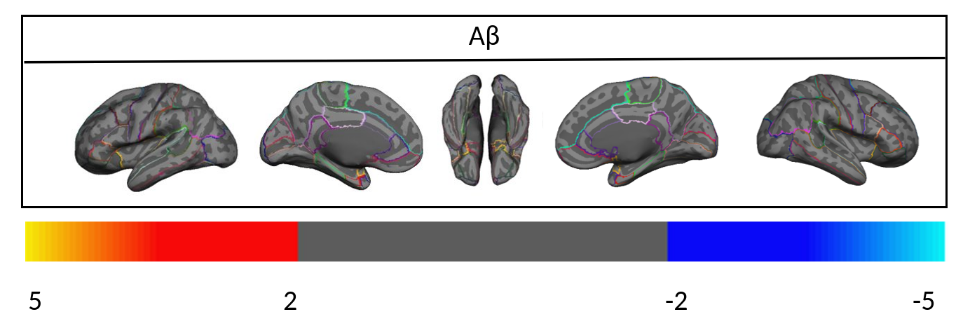


Fig. S3: Vertex-wise statistical map (t-value) of association between global Aβ pathology and cortical thickness throughout the entire cerebral cortex obtained in young subjects. The t-value at each vertex is color-coded with red to yellow colors representing increasing positive t-values and blue to light blue representing decreasing negative t-values and overlaid on the semi-inflated cortical surface of the MNI152 template. The association between global Aβ pathology and cortical thickness was not corrected for multiple comparison correction.


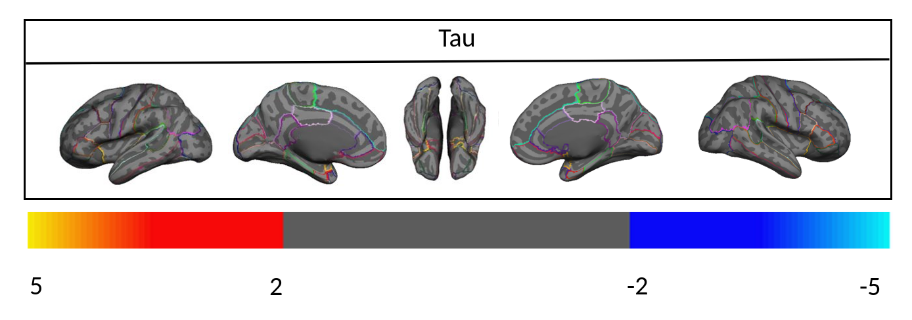


Fig. S4: Vertex-wise statistical map (t-value) of association between global tau pathology and cortical thickness throughout the entire cerebral cortex obtained in young subjects. The t-value at each vertex is color-coded with red to yellow colors representing increasing positive t-values and blue to light blue representing decreasing negative t-values and overlaid on the semi-inflated cortical surface of the MNI152 template. Association between global tau pathology and cortical thickness was not corrected for multiple comparison correction.


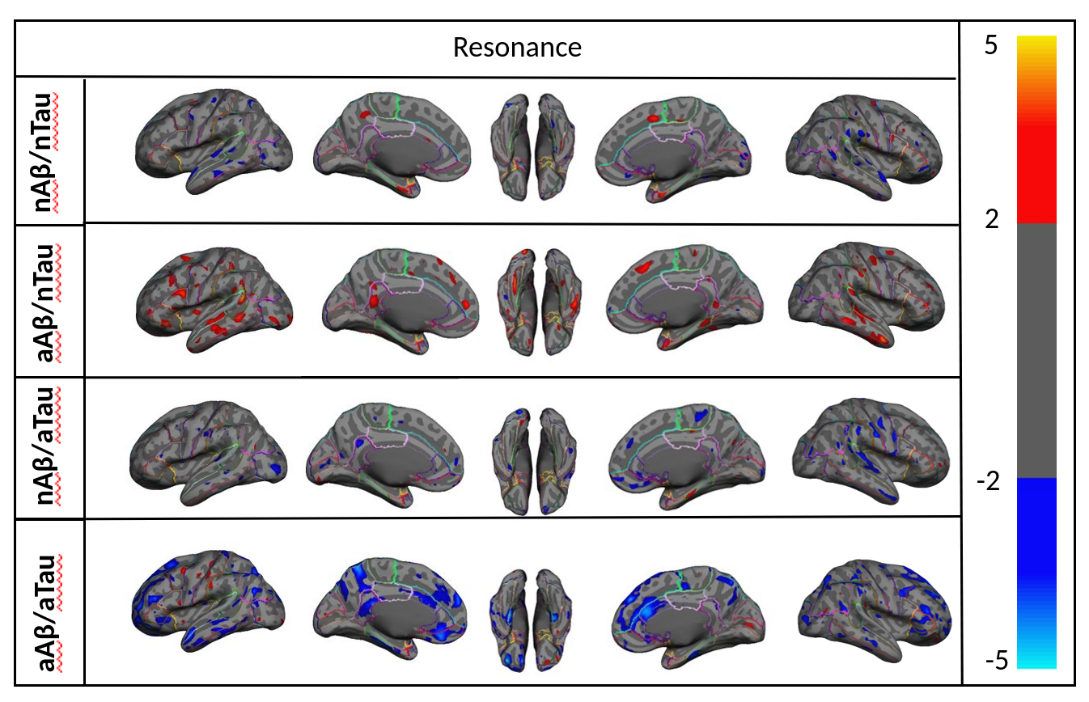


Fig. S5: Vertex-wise statistical map (t-value) of the association between resonance and cortical thickness throughout the entire cerebral cortex obtained in four categories of participants in the aAβ/aTau group. The t-value at each vertex is color-coded with a heatmap where red to yellow colors represents increasing positive t-values and blue to light blue represents decreasing negative t-values overlaid on the semi-inflated cortical surface of the MNI152 template. Associations between resonance and cortical thickness were not corrected for multiple comparison correction.


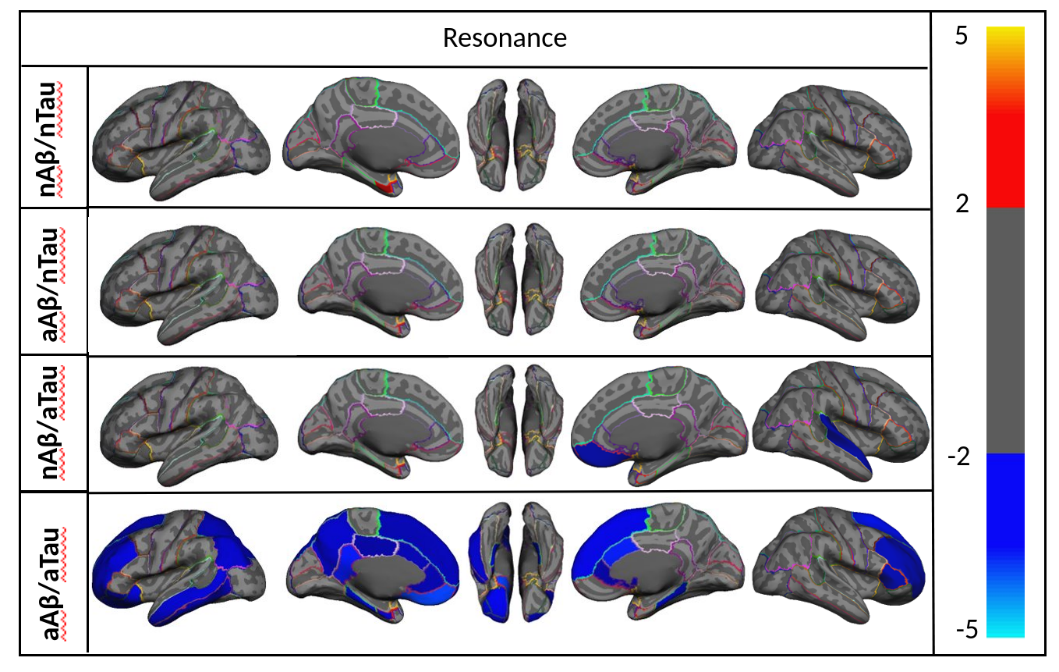


Fig. S6: (a) Region-wise statistical map (t-value) of the association between resonance and cortical thickness throughout cortical 68 ROIs obtained in the aAβ/aTau group. The t-value at each region is color-coded with a heatmap where red to yellow colors represents increasing positive t-values and blue to light blue represents decreasing negative t-values overlaid on the semi-inflated cortical surface of the MNI152 template. Association between resonance and cortical thickness was not corrected for family-wise errors.
